# Subpial delivery of adeno-associated virus 9-synapsin-caveolin-1 (*AAV9-SynCav1*) preserves motor neuron and neuromuscular junction morphology, motor function, delays disease onset, and extends survival in hSOD1^G93A^ mice

**DOI:** 10.7150/thno.72614

**Published:** 2022-07-11

**Authors:** Shanshan Wang, Taiga Ichinomiya, Paul Savchenko, Dongsheng Wang, Atsushi Sawada, Xiaojing Li, Tiffany Duong, Wenxi Li, Jacqueline A. Bonds, Eun Jung Kim, Atsushi Miyanohara, David M. Roth, Hemal H. Patel, Piyush M. Patel, Takahiro Tadokoro, Martin Marsala, Brian P. Head

**Affiliations:** 1 Department of Anesthesiology, VA San Diego Healthcare System, San Diego, CA, USA; 2 Department of Anesthesiology, University of California San Diego, La Jolla, CA, USA; 3 Neuroregeneration Laboratory, Department of Anesthesiology, University of California San Diego, La Jolla, CA, USA; 4 Department of Anesthesiology, University of the Ryukyus, Okinawa, Japan; 5 Department of Anesthesiology, Sapporo Medical University School of Medicine, Sapporo, Japan; 6 Department of Anesthesiology and Pain Medicine, Yonsei University College of Medicine, Seoul, South Korea; 7 Department of Anesthesiology, Nagasaki University Hospital, Nagasaki, Japan

**Keywords:** caveolin-1, membrane/lipid raft (MLRs), gene therapy, hSOD1^G93A^, amyotrophic lateral sclerosis, motor neuron, neuromuscular junction

## Abstract

Elevating neuroprotective proteins using adeno-associated virus (AAV)-mediated gene delivery shows great promise in combating devastating neurodegenerative diseases. Amyotrophic lateral sclerosis (ALS) is one such disease resulting from loss of upper and lower motor neurons (MNs) with 90-95% of cases sporadic (SALS) in nature. Due to the unknown etiology of SALS, interventions that afford neuronal protection and preservation are urgently needed. Caveolin-1 (Cav-1), a membrane/lipid rafts (MLRs) scaffolding and neuroprotective protein, and MLR-associated signaling components are decreased in degenerating neurons in postmortem human brains. We previously showed that, when crossing our SynCav1 transgenic mouse (TG) with the mutant human superoxide dismutase 1 (hSOD1^G93A^) mouse model of ALS, the double transgenic mouse (SynCav1 TG/hSOD1^G93A^) exhibited better motor function and longer survival. The objective of the current study was to test whether neuron-targeted Cav-1 upregulation in the spinal cord using *AAV9-SynCav1* could improve motor function and extend longevity in mutant humanized mouse and rat (hSOD1^G93A^) models of familial (F)ALS.

**Methods:** Motor function was assessed by voluntary running wheel (RW) in mice and forelimb grip strength (GS) and motor evoked potentials (MEP) in rats. Immunofluorescence (IF) microscopy for choline acetyltransferase (ChAT) was used to assess MN morphology. Neuromuscular junctions (NMJs) were measured by bungarotoxin-a (Btx-a) and synaptophysin IF. Body weight (BW) was measured weekly, and the survival curve was determined by Kaplan-Meier analysis.

**Results:** Following subpial gene delivery to the lumbar spinal cord, male and female hSOD1G93A mice treated with SynCav1 exhibited delayed disease onset, greater running-wheel performance, preserved spinal alpha-motor neuron morphology and NMJ integrity, and 10% increased longevity, independent of affecting expression of the mutant hSOD1G93A protein. Cervical subpial SynCav1 delivery to hSOD1G93A rats preserved forelimb GS and MEPs in the brachial and gastrocnemius muscles.

**Conclusion:** In summary, subpial delivery of SynCav1 protects and preserves spinal motor neurons, and extends longevity in a familial mouse model of ALS without reducing the toxic monogenic component. Furthermore, subpial SynCav1 delivery preserved neuromuscular function in a rat model of FALS. The latter findings strongly indicate the therapeutic applicability of SynCav1 to treat ALS attributed to monogenic (FALS) and potentially in sporadic cases (i.e., SALS).

## Introduction

Amyotrophic lateral sclerosis (ALS) derives its name from denervational atrophy of the muscles in the tongue, oropharynx, and limbs (***amyotrophy***), degenerating corticospinal axons resulting in thinning and scarring (***sclerosis***) of the ***lateral*** spinal cord, and thinning of the ventral roots. ALS is a progressive, paralytic disease due to loss of motor neurons (MN) in the brain and spinal cord. ALS signs include diffuse muscle weakness, fasciculations (spontaneous muscle twitching), hyperreflexia (upper), and paralysis (lower), and ultimately reducing life expectancy to 2-5 years 1, 2. In addition to these motor-associated pathologies, 15-20% of ALS cases also exhibit cognitive deficits as a result of degeneration of the frontal and temporal lobes (frontotemporal dementia or FTD) 3. Incidence of ALS is 3-5 per 100,000 persons globally and is present in both dominantly inherited familial (FALS, 5-10%) and sporadic (SALS, 90-95%) forms 2. Typical age-of-onset is 58-63 years and 47-52 years for SALS and FALS, respectively, with both being phenotypically similar. Unfortunately, the current FDA-approved treatments for ALS including Rilutek (riluzole, a glutamate antagonist) 1 and Radicava (edaravone, a free radical scavenger) 4 only provide limited extension of life; the former acts by attenuating excessive motor neuron firing and the latter works by decreasing oxidative stress.

For FALS, genetic interventions focused on knocking down key genes such as mutant form of super oxide dismutase 1 (*SOD1*) 5, however, due to the lesser-known etiology of SALS, a successful therapy for ALS will most likely require combinatorial neuroprotective interventions. What is known about ALS, including FALS and SALS, is that they are highly complex, heterogeneous neuropathological conditions resulting from motor neuronal loss due to a highly neurotoxic environment 6-10. Therefore, interventions that provide neuroprotection may preserve motor neuron survival and neuromuscular function even in the presence of known monogenic mutations or unknown sporadic etiology.

Previously we showed that neuron-targeting of the membrane/lipid raft (MLR) protein caveolin-1 (using a synapsin-driven Cav-1 engineered construct termed *SynCav1*) promotes hippocampal synaptic and neuroplasticity 11, significantly improves learning and memory in aged mice, and preserves learning and memory in an Alzheimer's disease (AD) mouse model independent of reducing amyloid plaques 12. Moreover, when crossing our SynCav1 transgenic mouse (TG) with the hSOD1^G93A^ mouse model of ALS, we observed preserved body weight (BW), longer survival, better running wheel (RW) performance, preserved spinal cord α (alpha) motor neurons, and more MLR-associated neurotrophin receptors (NTRs) in the spinal cord. In terms of human clinical relevance, a seminal finding from the United Kingdom (University of Sheffield) revealed decreased Cav-1 protein expression and disrupted MLRs in samples from sporadic human ALS cases due to mutations in the Cav-1 DNA enhancer region, suggestive of a novel ALS-associated risk variant 13.

One of the major challenges of treating neurodegenerative diseases, including ALS and AD, is to find the most effective manner by which to deliver vectors while minimizing the off-target effect. Although some have demonstrated penetration into the central nervous system and spinal cord using intrathecal or intraventricular delivery using high titers of genetic material, which unfortunately limits the effectiveness and safety of using AAV9-based vectors to treat afflicted spinal cord and brain tissue in ALS 14-17. To achieve broader tissue distribution with greater efficacy, the current study utilized the novel spinal subpial (SP) vector delivery route developed by the Marsala laboratory 14.

Over the past few years, the predominant approach to treat ALS is to target known monogenic causes of FALS 15, 18-23. Instead of targeting the genes or proteins attributed to the gain of toxicity, the present study tested the hypothesis that subpial delivery of neuroprotective *AAV9-SynCav1* to the spinal cord of hSOD1^G93A^ mice may mitigate disease progression, protect motor neurons, maintain neuromuscular junctions (NMJs), and extend survival, independent of targeting mutant hSOD1^G93A^ protein. We now show for the first time that a one-time subpial delivery of *AAV9-SynCav1* to hSOD1^G93A^ mice at lumbar section significantly extends longevity and preserves BW in both male and female mice, preserves lumbar motor neuron morphology and NMJ morphology in the lower limb muscle, and maintains better RW performance independent of affecting expression of mutant hSOD^G93A^protein. This study further extends the neuroprotective and therapeutic potential of *SynCav1* gene therapy to another distinct neurodegenerative condition (i.e., ALS), irrespective of disease etiology, and thus *SynCav1* has the potential to treat a variety of neurodegenerative conditions attributed to either familial or sporadic forms. Furthermore, *SynCav1* gene therapy may work synergistically in combination with other interventions (e.g., antisense oligonucleotides, antibodies, small molecules) that specifically target monogenic familial forms of ALS and other neurodegenerative conditions.

## Materials and Methods

### Animal

Human mutant superoxide dismutase-1 (hSOD1^G93A^)-transgenic (Tg) and C57BL/6 mice were purchased from Jackson Laboratory (Bar Harbor, ME, USA) and bred in-house. Wild type (WT) littermates were used as control. All animal protocols were approved by the Veterans Administration San Diego Healthcare System Institutional Animal Care and Use Committee (#A20-030). Mice were reared (3-5/cage) with free access to food and water. WT, and hSOD1^G93A^ mice were allocated to 4 groups randomly: WT, hSOD1^G93A^ naïve (no surgery), hSOD1^G93A^ + control (subpial surgery with same volume of *AAV9-SynNull* or saline), and hSOD1^G93A^ + *SynCav1* to receive subpial lumbar vector injections. Spinal cord tissue was processed for biochemistry, histopathology, and immunofluorescence at the indicated time points.

Sprague-Dawley rats were treated in compliance with the Guide for the Care and Use of Laboratory Animals (National Institutes of Health) in the Marsala lab and approved by UCSD. Transgenic rats expressing ALS-linked human SOD1 mutant protein (hSOD1^G93A^) exhibit an ALS-like neurodegenerative phenotype characterized by diminished body weight from muscle atrophy, weakness/paralysis, fasciculations, and early death, along with reductions in functional cortical alpha motor neurons and ventral horn alpha motor neurons that can be observed histologically 24. The hSOD1^G93A^ transgenic male rats (Marsala lab, UCSD) were bred with WT female rats to produce WT and hSOD1^G93A^ positive male rats.

### hSOD1^G93A^ Genotyping

hSOD1^G93A^ mice and rats were confirmed by genomic DNA extraction and PCR using the Qiagen DNeasy Blood and Tissue Kit (69504; Qiagen, Valencia, CA, USA). PCR was performed for the hSOD1 genes by using the following protocol: denaturation at 94 °C for 2 min, followed by 10 cycles at 94 °C for 20 s, 65 °C for 15 s (- 0.5 per cycle decrease), and 68 °C for 10 s, then followed by 28 cycles at 94 °C for 15 s, 60 °C for 15 s, 72 °C for 10 s, then 72 °C for 2 min and hold at 10 °C. All primers were purchased from Integrated DNA Technologies (Coralville, IA, USA). hSOD1G93A-positive mice were confirmed using the following primers: primer 1, hSOD1 Forward [CAT CAG CCC TAA TCC ATC TGA (oIMR0113)]; primer 2, hSOD1 Reverse [CGC GAC TAA CAA TCA AAG TGA (oIMR0114)]; primer 3, Internal control Forward [CTA GGC CAC AGA ATT GAA AGA TCT (oIMR7338)]; and primer 4, Internal control Forward [GTA GGT GGA AAT TCT AGC ATC ATC C (oIMR7339)]. These primers were used to detect a product size of 800 for mouse endogenous SOD1 and 600 base pair (bp) for human SOD1 as previously described 25. Transgenic rats were genotyped by PCR amplification for the hSOD1^G93A^. PCR products were separated on a 1% agarose gel (35 min at 135 volts).

### Viral vectors and *SynCav1* Construct

A self-complementary AAV (scAAV) construct expressing neuron specific synapsin (Syn) promoter with the Cav-1 cDNA was generated at the UCSD Viral Vector core as previously described 12. To link the neuron specific synapsin (Syn) promoter with the Cav-1 cDNA, an XbaI-SalI DNA fragment containing the Syn promoter was inserted into the NheI-SalI sites of pEGFP-N1 (Clontech). The resulting plasmid was designated pSyn-EGFP. A 537 bp Cav-1 cDNA was isolated from the pCRII-TOPO vector (Invitrogen) by digestion with PmeI-NotI and inserted into the SmaI-NotI site of the pSyn-EGFP to generate pSyn-Cav-1, in which the EGFP gene was replaced with Cav-1 cDNA. The Syn-promoter-Cav-1 cassette was isolated from pSyn-Cav-1. The scAAV vector construct, expressing Cav-1 or RFP driven by the Syn promoter, was made by DNA synthesis of the Syn promoter (480 bp) through Cav-1 (537 bp) or enhanced RFP (700 bp) and cloning it into an scAAV backbone plasmid. The *scAAV9-SynCav1, scAAV9-SynRFP*, or *scAAV9-SynNull* vectors were produced by transient transfection of HEK293T cells with each vector plasmid, pRep2/Cap9 and pAd-Helper plasmid. Helper virus-free AAV vectors were produced by Polyethylenimine (PEI)-mediated transient co-transfection of HEK293T cells with three plasmids [pVector, pRep2/CapX (X stands for each serotype) and pAd-Helper]. Cells were collected at 72 hrs post-transfection and cell lysates were made by 3x freeze/thaw cycle. AAV vectors in the cell lysates were purified by combination of sucrose-cushion ultracentrifugation and anion-exchange column chromatography. Virus titers were measured by real-time Q-PCR to determine genome copy (g.c.) number in the vector preparations (g.c./ml) as a measure of AAV particles with full genome content. Final virus titers for all three vectors are ~2.0 x10^13^ g.c./ml*.*

### Subpial surgery and AAV administration

All surgical procedures were done under sterile conditions and approved by the local IACUCs. *Mouse subpial procedure*: Lumbar (L1-L3) subpial vector injection in mice was performed as previously described 14. Briefly, mice were anesthetized with 2-3% isoflurane, the surgical area was shaved and cleaned, and a skin incision was made at the thoracic 12 (T12)-L1 vertebral level. Under the dissecting microscope, a dorsal laminectomy of the thoracic (T13) vertebrae was performed, and the dura overlying the lumbar (L1) spinal segments was cut open using a 31- gauge (G) needle. The pia mater was then punctured using a 36-G pia-penetrating needle, followed by the insertion of a blunt 36-G injection needle containing the scAAV9 vector. Both the pia-penetrating and the subpial injection needles were mounted on fine XYZ manipulator with needle holder (SM-15HE, Narishige, Japan). *Rat subpial procedure*: Wild type (WT) or hSOD1^G93A^ Sprague Dawley rats were anesthetized by isoflurane with 2% in room air and placed into a Lab Standard Stereotaxic frame (Stoelting, Wood Dale, IL). Rats were elevated 3 cm by placing on a plastic box. A homeothermic heating blanket which was set at 37 °C with feedback from a rectal thermometer (Harvard Apparatus, Holliston, MA) was used to maintain body temperature of rats during the procedure. Rats were then placed in Spine Adaptors (Stoelting, Wood Dale, IL) and a C4-5 laminectomy was performed to expose the dorsal surface of spinal segment C4-5 using an air-powered dental drill and binocular microscope. The dura and pia mater were carefully incised using a 27- G needle and a polyethylene tubing catheter, of which its size was 0.008 ID x 0.02 OD, (SAI infusion technologies, Lake Villa, IL) was inserted into the subpia-space. Injections were made using 100-μL Hamilton Gas Tight syringe (Hamilton, Reno, NV). Mice received a total of 10 μl to the lumbar spinal cord and rats received a total of 20 μl of *AAV9-SynCav1* (~2.0 x 10^13^ genome copies (g.c.)/ml) vector to the cervical spinal cord by subpial delivery (5 μl bilateral to the lumbar spinal cord, 10 μl total over 600 s for mice; 10 μl bilateral to the cervical spinal cord, 20 μl total over 600 s for rats) using a 50 μl Hamilton syringe and digital infusion pump (Microinjector MINJ-PD, Tritech Research). After vector delivery, the muscle and skin were closed using 7/0 Prolene and suture clips. Before recovery from anesthesia, the animals received subcutaneous fluids, antibiotics (cefazolin sodium 50 mg/kg) and the initial dose of pain medication (slow release Buprenex 0.05 mg/kg). Control group received laminectomy and subpial injection of or equal volume of either *AAV9-SynNull* or sterilized saline.

### Assessment of disease onset and survival

Disease onset and late disease were retrospectively defined as the first sign of weight loss from peak body weight and 10% weight decline from peak body weight, respectively 18, 26, 27. Survival analyses were performed using Kaplan-Meier analyses as previously described 25. Survival endpoint is defined by the inability of the mouse to adjust itself within 30 seconds (s) when placed on its back, a humane endpoint that guarantees that animals are euthanized before they are unable to eat or drink.

### Voluntary running wheel (RW)

At 8 and 16-week-age, mice were housed individually in plastic cages measuring 40 x 40 x 35 cm (length x width x height) with a 11.5 cm diameter running wheel mounted on the cage side (SuperFlex Open Field, Omnitech Electronics, Inc., Ohio, USA). The mice had free access to food and water ad libitum and were checked daily for health and wellness. The mice had open access to running wheels under conditions of a 12 h light/dark cycle (lights off from 6 AM to 6 PM). The duration of each experiment was 36 h including 2 dark cycles. The mice will be placed in the cages at least 24h prior to data collection. A sensory beam, connected to a desktop computer via a software (Fusion v6.5 r9999 SuperFlex Edition, Omnitech Electronics, Inc., Ohio, USA), was used to record wheel rotations. Running velocity (m/min), and total distance (m) were used for analysis.

### Motor Evoked Potential (MEP)

Rats were anesthetized by intramuscular injection of ketamine (100 mg/kg) for motor-evoked potentials (MEP) recordings in the branchial or gastrocnemius muscles. Two 30-G stimulating electrodes were placed subcutaneously overlying the left and right motor cortex. In all rats, MEP of upper forelimb (brachial) and lower limb (gastrocnemius) were recorded from the muscles elicited by transcranial electrical stimulation with a pulse duration of 200 μs at 15 mA. Recording electrodes were connected to an active head stage (Warner Instruments, Hamden, CT), and the recorded signal is amplified using DP-311 differential amplifier (Warner Instruments, Hamden, CT). The amplified signal was acquired by the PowerLab 8/30 data acquisition system (ADInstruments, Australia) at sampling frequency of 20 kHz, digitized and stored using LabChart 7 (ADInstruments, Australia) in PC for analysis.

### Grip Strength

Cohorts of age-matched transgenic rats and littermate controls were tested for loss of forelimb grip strength by using a grip strength meter (AMETEK, Inc., Berwyn, PA) at 8 wk (before subpial-injection) and weekly from 10 to 20 wk. Rats were held in front of a horizontal bar, such that only the forelimb paws were able to grasp the bar. The rats were gently pulled back with steady force until both paws released the bar. Each rat was given 6 attempts and the mean grip strength was recorded for analysis.

### Immunofluorescence (IF) Microscopy

Motor neuron morphology and Cav-1 target validation was assessed by IF. Specifically, mice were perfused with saline and the spinal cord was dissected followed by 48 hrs post-fix in z-fix (ANATECH, #170) and dehydration in 30% sucrose. 30 µm thickness coronal spinal cord section was then incubated with Alexa Fluor 488 conjugated anti-choline acetyltransferase (ChAT) (abcam, ab225263, 1:400) and Alexa Fluor 594 conjugated caveolin-1 antibody (7C8) (Novus biologicals, NB100-615AF594, 1:1000) for overnight at 4 °C. Nuclei were counterstained with antifade DAPI mounting medium (ProLong Gold antifade mounting medium with DAPI, P36931; Invitrogen, Waltham, MA, USA). A minimum of three coronal spinal cord sections per mouse were used for quantitation of motor neuron.

For NMJ analysis, 30 µm thickness horizontal gastrocnemius muscle section was stained with 488-conjugated bungarotoxin-a (Btx-a) (B13422; 1:250; Invitrogen) to label post-synaptic structure and synaptophysin (ab32594; 1:200, abcam) to label pre-synaptic vesicles followed by 1 h incubation of secondary (Alexa Fluor 568, 1:200; B13422, Invitrogen, Waltham, MA, USA) at room temperature. Occupancy of synaptophysin labeled presynaptic structure in Btx-a labeled postsynaptic was analyzed to assess NMJ morphology 28. Slides were imaged using Keyence All in One microscopy (BZ-X700, Keyence corporation of America, IL, USA) and confocal laser microscopy (A1; Nikon, Tokyo, Japan), Z stack with 10um was taken and projected to a single image for analysis.

### Biochemical Characterization of Membrane/Lipid Rafts (MLRs) and Immunoblot (IB) assay

Thoracic (T12) to lumbar (L2) spinal cord tissue were homogenized at 4 ºC in 500 mM sodium carbonate (pH 11.0) (containing protease and phosphatase inhibitors) and then sonicated 3x for 15 s. Samples (0.5 mg/ml) were subjected to sucrose density gradient fractionation as previously described 29. Homogenates and fractions were immunoblotted using primary antibodies Cav-1 (Cell Signaling #3268; 1:1000), B8H10 (Medimabs MM-0070-P, 1:500), ChAT (Millipore, AB144P, 1:1000), and GAPDH (Cell Signaling #2118s; 1:1000) overnight at 4 ºC followed by incubation with IR-dye labeled secondary antibody for 1 hour. Membranes were scanned by LiCor Odyssey followed by densitometric analysis.

### Statistical analysis

Sample size is indicated under each section of the Methods and in the figure legends. Before determining statistical significance, data were checked for normal distribution. Data were analyzed by Student's t test, one-way analysis of variance (ANOVA) or 2-way ANOVA followed by Fisher's LSD, Tukey's, Šídák's, or Bonferroni's multiple comparisons post hoc analyses as appropriate using Prism 7 software (GraphPad, La Jolla, CA, USA). The Kaplan-Meier survival curve was analyzed using a log-rank test. Mean and median survival were analyzed by an unpaired Student's t-test. Significance was assumed when P < 0.05. All experimental groups were blinded, and code was broken only for analysis. For correlation analysis, a simple linear regression with 95% confidence intervals was performed.

## Results

### *AAV9-SynCav1* delivery to the lumbar spinal cord increases Cav-1 protein expression in the lumbar and thoracic cord

**Figure 1:**
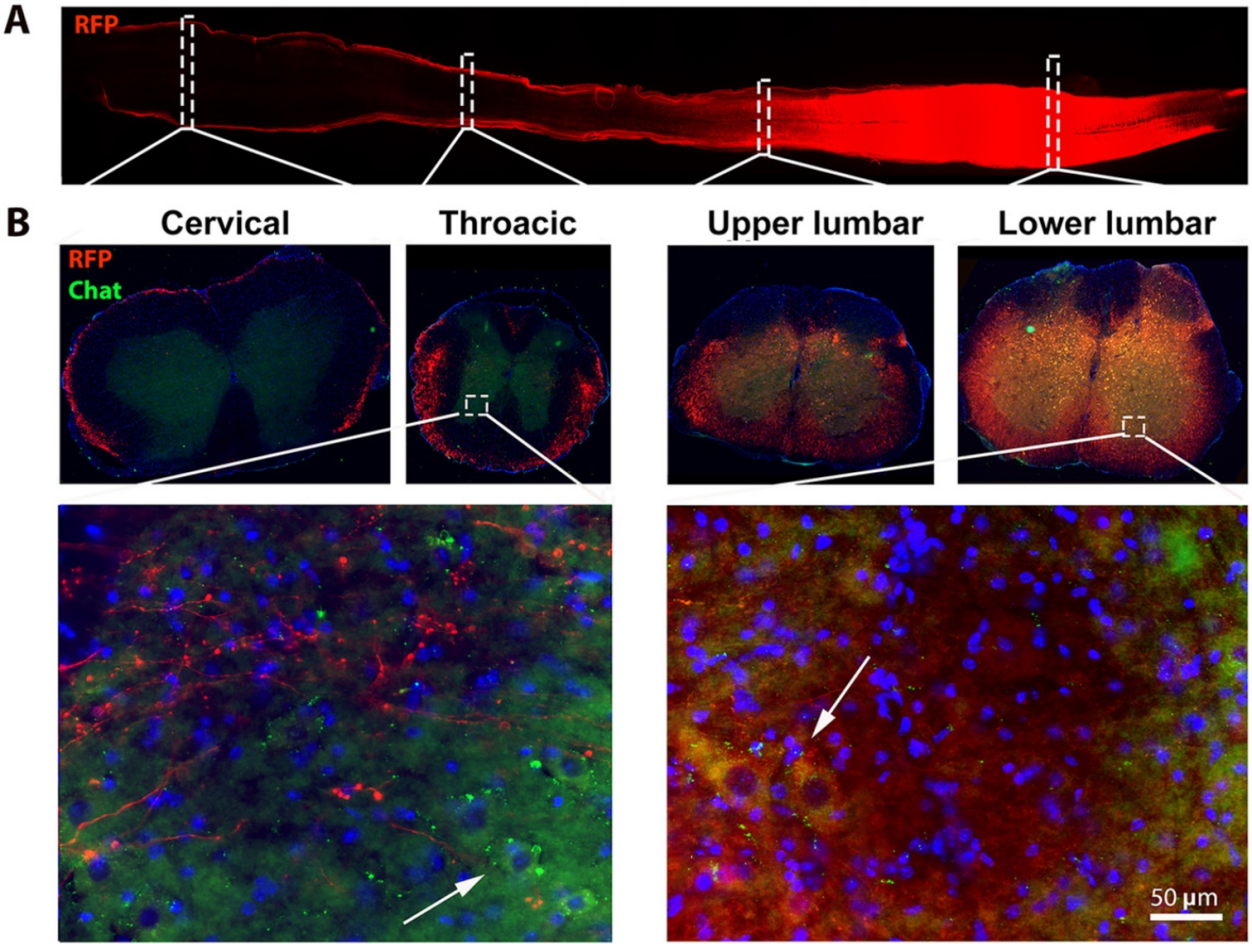
** Subpial *AAV9-SynRFP* delivery to the lumbar spinal cord (L1) resulted in RFP transgene expression across entire lumbar cords.** Spinal cords were collected 30 days post-AAV9-*SynRFP*. (A) Horizontal section of spinal cords showed strong RFP expression around lumbar cords. (B) Coronal sections of spinal cord were stained with motor neuron marker ChAT. Representative images showed strong RFP expression in the lumbar segment including ChAT positive lower motor neuron at the ventral horn area, while cervical and thoracic exhibit weak RFP signal predominantly in white matter.

Previous work from other groups have reported that the occurrence of symptomatic onset varies from 8 to 12 weeks (wk) 30 in the hSOD1^ G93A^ mice using different methods and parameters 31, 32. For example, motor deficits and motor unit loss in lower limb muscle due to selective preferential vulnerability of large, fast motor units, innervated by large motor neurons was observed in 8 wk hSOD1^G93A^ mice, several weeks prior to the standard measurable onset of overt clinical symptoms in this mouse model 33-35. To achieve effective AAV9 transduction of spinal cord motor neurons and peak expression of the Cav-1 transgene (which takes approximately 3-4 wk post AAV delivery), we chose to deliver *AAV9-SynCav1* at week 8 via subpial delivery, a highly effective route of administration used to accomplish effective strong transgene expression in the mammalian spinal cord 11, 12. As shown in Figure 1, lumbar subpial delivery of *AAV9-SynRFP* resulted in a strong expression of RFP in the lower thoracic up to lumbar (T11 to L5), with minimal expression detected in upper thoracic and cervical regions. Our previous study demonstrated that SynCav1+/ hSOD1^G93A^+ transgenic mice exhibit slowing of disease progression, we therefore initially performed a dose-dependent study for subpial *AAV9-SynCav1* delivery to determine the optimal dose necessary to achieve Cav-1 protein expression in the spinal cord similar to that measured in SynCav1+ transgenic mice 25. As shown in Figure S1, the optimal dose of *AAV9-SynCav1* to achieve 4-fold Cav-1 overexpression in the thoracic and lumbar spinal cord was 2 x 10^11^ total g.c. (10 μl of 2 x 10^13^ g.c./ml). This dose was used in all the following studies.

### *AAV9-SynCav1* significantly delays BW loss and disease onset, and extends survival in hSOD1^G93A^ mice

**Figure 2:**
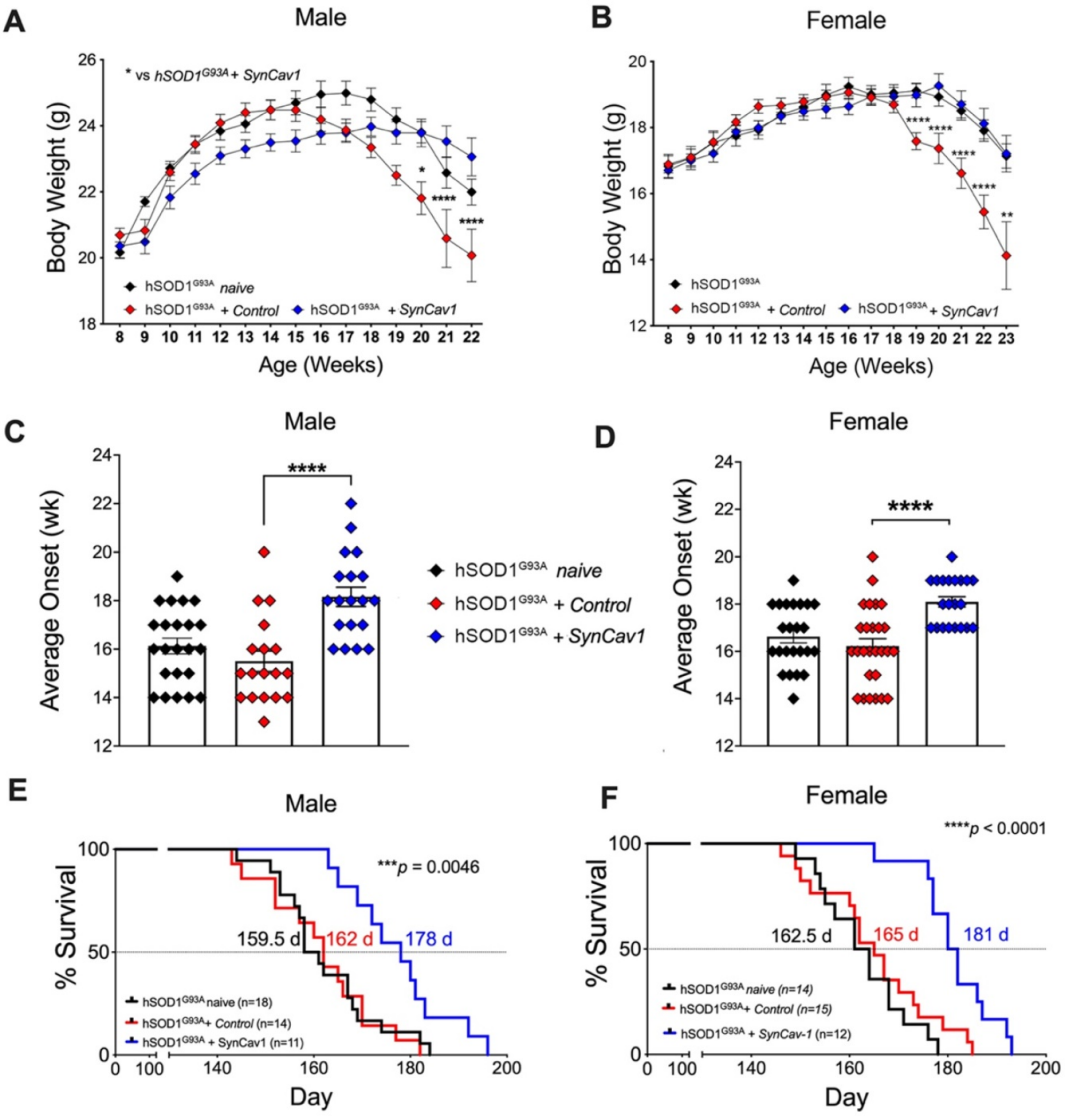
***AAV9-SynCav1* subpial delivery significantly delays disease onset, preserves BW and extends survival in hSOD1^G93A^ mice.** (A-B) Body weight (BW), (C-D) Average disease onset timepoint (defined by peak BW) and (E-F) Kaplan-Meier survival curve for male and female mice. Groups: hSOD1^G93A^ naïve (no surgery), hSOD1^G93A^ + control and hSOD1G93A + *SynCav1* mice. BW was expressed as mean ± SEM, Two-way ANOVA with Šídák's multiple comparisons test was used for A and B. One-way ANOVA with Dunnett's multiple comparison test was used for C and D (all compared to hSOD1^G93A^ + control group). Kaplan-Meier Curve was expressed as median days (d) survival and analyzed using a Mantel-Cox Log-rank test (***p < 0.005, ****p < 0.0001).

We next assessed whether subpial delivery of *AAV9-SynCav1* could provide therapeutic benefit to the hSOD1^G93A^ mouse model of ALS. As shown in Figure 2A and 2B, compared to hSOD1^G93A^ + control, hSOD1^G93A^ + *AAV9-SynCav1* mice showed significantly delayed BW loss in both sexes. As shown in Figure 2C and 2D, average disease onset of hSOD1^G93A^ + *AAV9-SynCav1* mice was 18 wk for male (vs 15 wk hSOD1^G93A^ + control male) and female (vs 16 wk hSOD1^G93A^ + control female), an approximately 15% delay compared to the control group. In terms of disease progression, late disease onset (i.e., 10% decrease from peak BW) 26 was measured at 20 wk for both male and female hSOD1^G93A^ + control mice, while *AAV9-SynCav1-*treated hSOD1^G93A^ female mice exhibited delayed late disease onset until 23 wk (~11% BW decline); *AAV9-SynCav1-*treated hSOD1^G93A^ male mice only exhibited ~4% BW decline at 22 wk (end of body weight record due to loss of multiple objects in the hSOD1^G93A^ + control groups).

In terms of survival, although subpial surgery had no effect on survival rates for hSOD1^G93A^ + control versus hSOD1^G93A^ naïve, we observed a greater decline in BW in the sham surgery control group compared to naïve hSOD1^G93A^ mice between 18-22 wk (Figure 2A and 2B), indicating that subpial surgery alone appeared to impact disease progression. Because of this effect caused by the subpial procedure alone, for all subsequent assays (RW, histological quantitation of MN and NMJ morphology), Student's t-test comparison was performed only within the non-surgery (i.e., WT vs hSOD1^G93A^ naïve) and the surgery group (i.e., hSOD1^G93A^ + control vs hSOD1^G93A^ *+ SynCav1*) to remove the confounding effects from subpial surgery on any measurable efficacy afforded by *SynCav1* gene delivery*.* As shown in Figure 2E-2F,* AAV9-SynCav1* gene delivery significantly extended survival in male and female (10%) hSOD1^G93A^ mice when compared to hSOD1^G93A^ + control mice (Figure 2E, median male survival days (d): hSOD1^G93A^ + *SynCav1* (178 d) vs hSOD1^G93A^ + control (162 d), ***p = 0.0046, Kaplan-Meier; n = 11-18; Figure 2F, median female survival: hSOD1^G93A^ + *SynCav1* (181 d) vs hSOD1^G93A^ + control (165 d); ****p < 0.0001, Kaplan-Meier; n = 12-15)*.* These findings are direct evidence that *SynCav1* significantly delays disease onset and extends longevity (i.e., slowing % BW loss) in hSOD1^G93A^ mice.

### Subpial *AAV9-SynCav1* delivery preserves Cav-1 localization to lumbar spinal cord MN plasmalemmal MLRs in hSOD1^G93A^ mice

**Figure 3:**
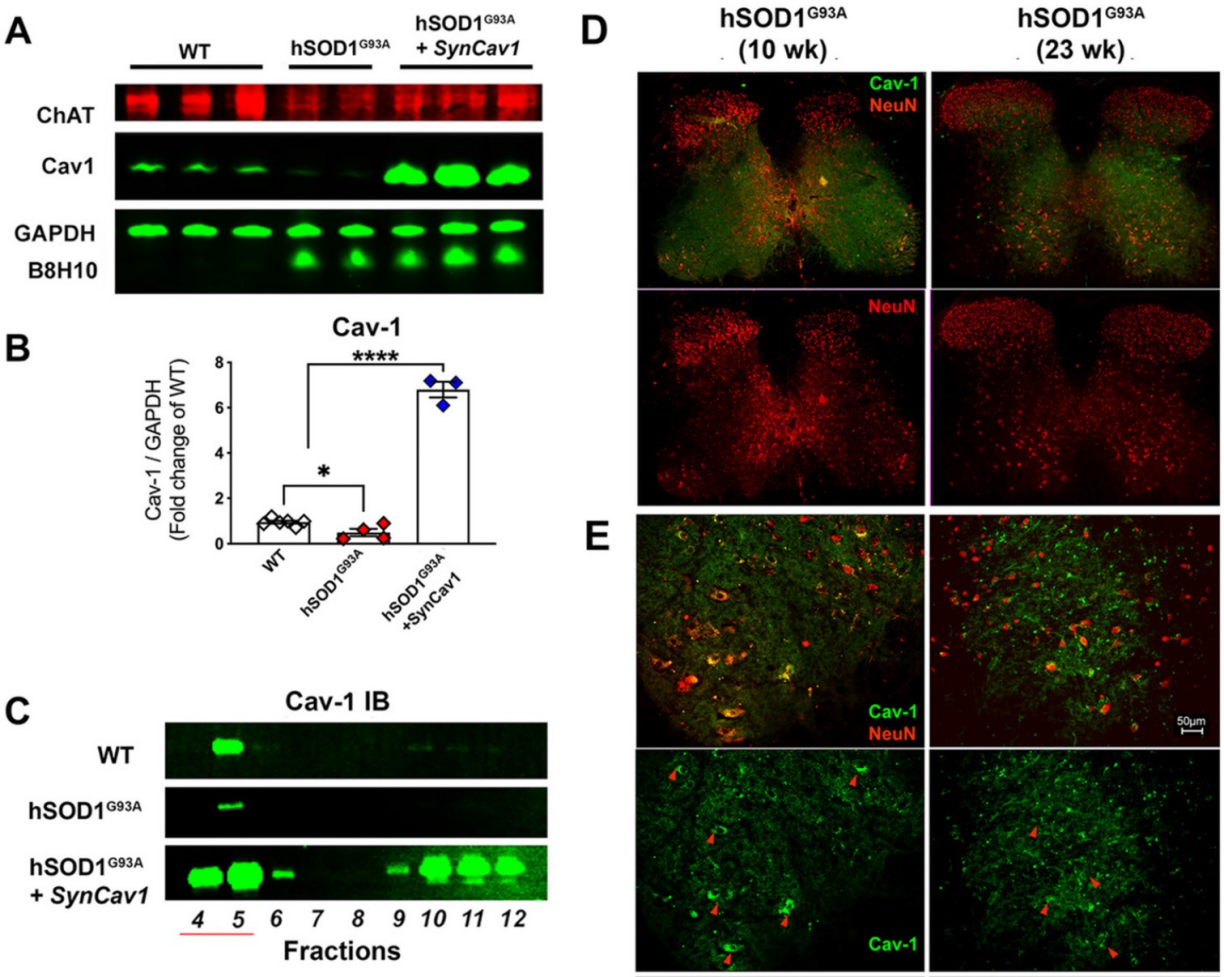
** Decreased neuronal Cav-1 expression in the lumbar spinal cord MN from end-stage hSOD1^G93A^ mice.** (A-B) Lumbar spinal cords from end-stage hSOD1^G93A^ mice (21-23 wk) were subjected to IB assays to measure Cav-1, ChAT (motor neuron marker), and mutant hSOD protein (B8H10). While Cav-1 expression was significantly decreased in the end-stage naïve hSOD1^G93A^ mice, subpial delivery of *AAV9*-*SynCav1* resulted a 6-8 folds increase of Cav-1 protein expression, with no significant change in expression of hSOD1G93A. (C) Representative IB of lumbar spinal cord homogenates showed decreased MLR-localized Cav-1 (i.e., buoyant fractions 4-5) in naïve hSOD^ G93A^ mice compared to WT and hSOD^ G93A^ + *SynCav1*. (D) IF microscopy was performed to measure Cav-1 and NeuN-positive MN in lumbar sections of 10 wk (early symptomatic) and 23 wk (end-stage) hSOD^ G93A^ mice. (E) Higher magnification of ventral horn area revealed polarized Cav-1 expression on large NeuN-positive MN cell bodies at 10 wk (red arrowheads in left panels). At 23 wk, both Cav-1 expression on NeuN-positive MN and the size of NeuN-positive MN cell bodies were decreased in the ventral horn region (arrowheads in right panels) from hSOD^ G93A^ mice. Data are presented as mean ± SEM. Data were analyzed using One-way ANOVA with Fisher's LSD multiple comparisons post hoc test. (n = 3-6; *p < 0.05). Scale bar, 50 μm.

As shown in Figure 3A-3B, IB assays of lumbar spinal cord tissue homogenates from terminal stage 23 wk hSOD1^G93A^ mice (terminal stage as defined by 162 d median survival in Figure 2C and 2D for non-treated hSOD1^G93A^ mice) showed elevated B8H10 (mutant and WT human SOD protein) and decreased Cav-1 expression. IB of MLR fractions revealed that Cav-1 was predominantly localized to MLR fractions 4-5 (Figure 3C) in normal WT mice, which is consistent with previously published work in brain and spinal cord tissue 11, 25, 29. However, Cav-1 expression decreased in MLR fractions from the lumbar spinal cords of 23 wk (end stage) hSOD1^G93A^ mice. Subpial *AAV9-SynCav1* delivery to 8 wk hSOD1^G93A^ mice significantly increased Cav-1 expression and subcellular localization of Cav-1 to MLR fractions, with no effect on expression of mutant SOD protein (B8H10). One limitation to IB assays is that when performed on whole spinal cord (or brain) tissue, one is incapable of distinguishing cell-specific protein changes (i.e., neuronal versus non-neuronal cells). To better elucidate the decreased Cav-1 expression as measured by IB, we additionally performed IF on lumbar spinal cord tissue from 10 wk (early symptomatic) and 23 wk (end stage) using neuronal marker NeuN and Cav-1 (Figure 3D-3E). While Cav-1 expression exhibited a polarized plasma membrane localization in NeuN-positive large MN cell bodies at the ventral horn of 10 wk hSOD1^G93A^ mice, a distribution pattern similar to WT mice (Figure 3E, red arrow heads); lumbar sections from 23 wk hSOD1^G93A^ mice confirmed that the decreased Cav-1 expression specifically occurred in ventral horn degenerated MN.

### *AAV9-SynCav1* treated hSOD1^G93A^ mice exhibit better RW endurance

**Figure 4:**
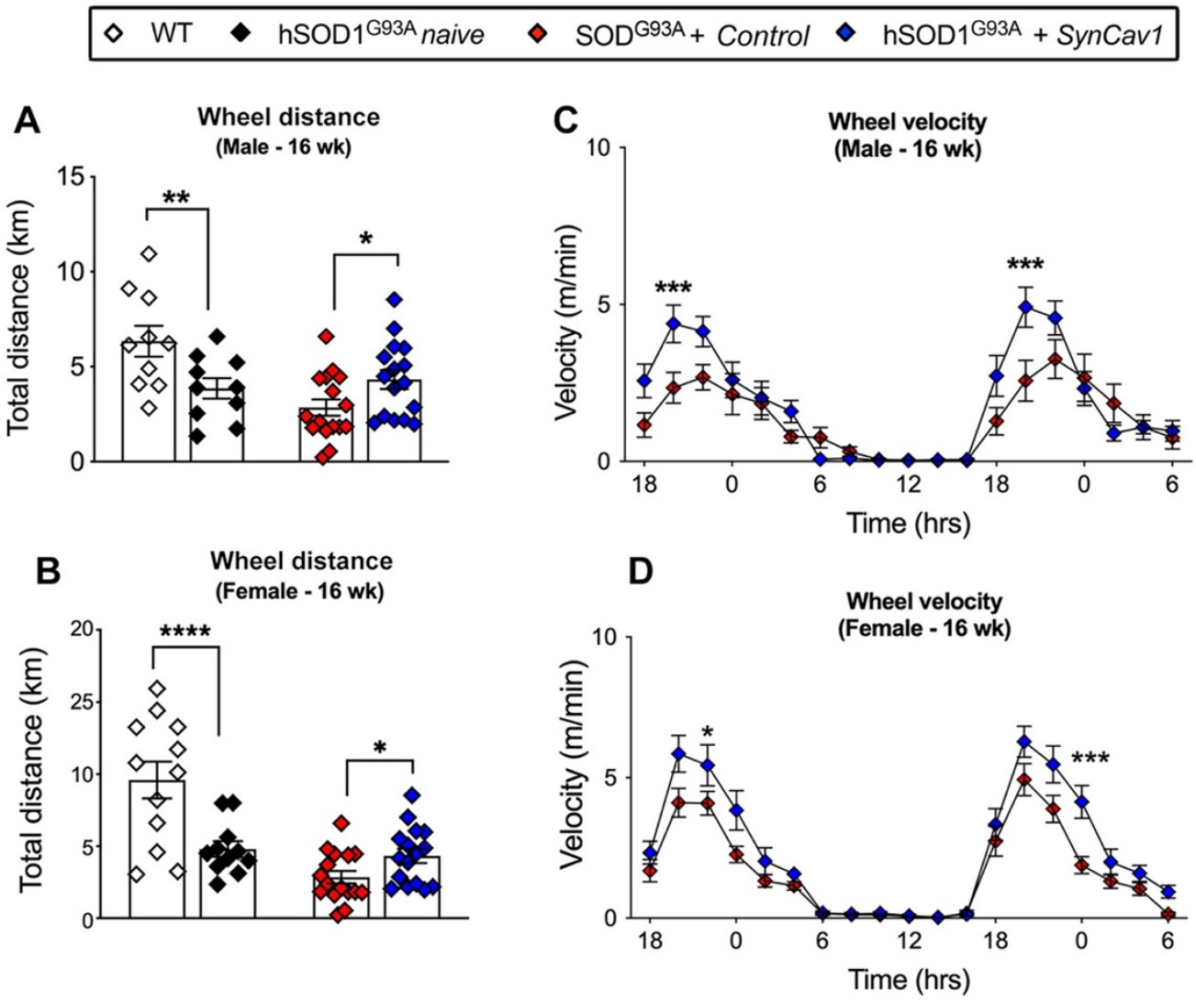
***AAV9-SynCav1* treated hSOD1^G93A^ mice perform better on voluntary running wheel (RW) motor test.** The 16 wk male and female total distance (km) (A, B) and mean RW velocity (m/s) (C, D) measured for 36 h. Data are expressed as means ± SEM. (n = 10-19/group). Student's t-test was used for A and B. 2-way ANOVA with Fisher's LSD multiple comparison test was used for C and D (*p < 0.05, **p < 0.01, ***p < 0.005, ****p < 0.0001).

At 8 wk (before subpial surgery), mice from all groups were trained for voluntary RW test. hSOD1^G93A^ mice were then randomly allocated to naïve, control and *SynCav1* groups. Motor function was assessed by voluntary RW test again at week 16. Consistent with our previous study, both sexes of hSOD1^G93A^ naïve mice ran significantly less total distance compared with WT (Figure 4A, male, *p = 0.02, Student's t-test, n = 10; Figure 4B, female, **p = 0.003 Student's t-test, n = 11-12). hSOD1^G93A^ + control mice ran slightly less than hSOD1^G93A^ naive with no significant difference between these two mice at 16 wk. hSOD1^G93A^ + *SynCav1* mice exhibited significantly greater total distance run compared with control mice (Figure 4A, male, **p = 0.03, Student's t-test, n = 10; Figure 4B, female, **p = 0.004 Student's t-test, n = 11-12). Furthermore, hSOD1^G93A^ +* SynCav1* mice exhibited significantly higher RW velocity compared with hSOD1^G93A^ + control mice (Figure 4C, male; Figure 4D, female; 2-way ANOVA, Fisher's LSD, n = 16-19)*.* These data demonstrate that subpial *AAV9-SynCav1* delivery preserved motor function as measured by RW performance.

### *AAV9-SynCav1* preserves alpha-motor neuron morphology in the spinal cord lumbar ventral horn and NMJ morphology in the gastrocnemius muscle of hSOD1^G93A^ mice

**Figure 5:**
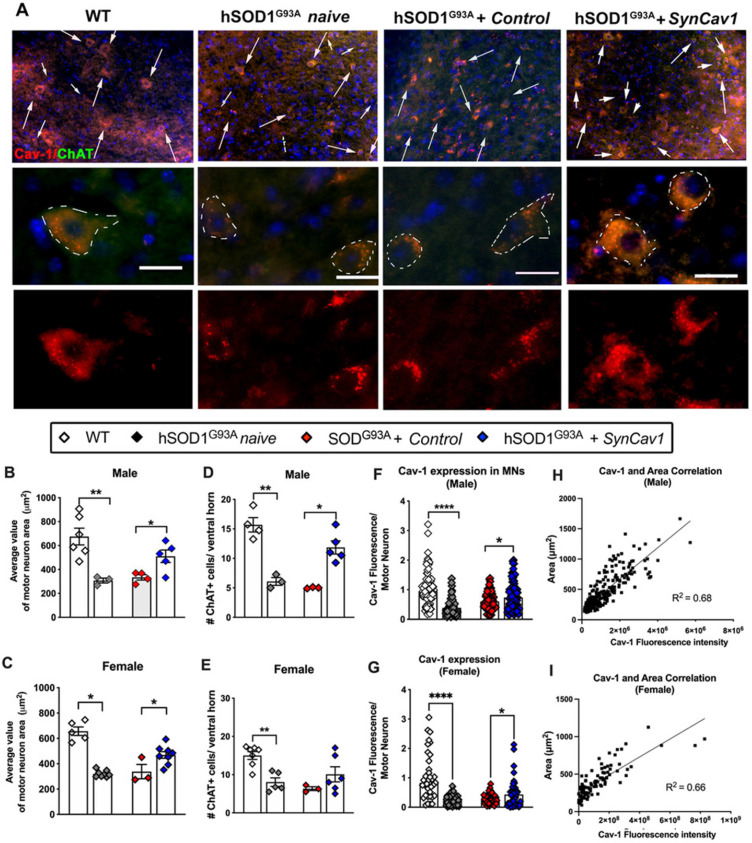
***SynCav1* prevented lumbar ventral horn motor neuron loss in 19 wk hSOD1^G93A^ mice*.*** (A) Motor neuron (MN) was identified by the MN marker choline acetyl transferase (ChAT) in the lumbar ventral horn. Scale bar, 20 μm. (B-E) Number and average cell body area (μm^2^) of MNs per animal. A minimal of 3 images was used to calculate the average value. (F-G) Cav-1 expression was measured in individual ChAT+ somas and normalized to its area. Data are presented as mean ± SEM. Student's t-test (n = 3-8 mice/group; *p < 0.05, **p < 0.01, ****p < 0.001). (H-I) Simple linear regression with Pearson's r correlation analysis was performed comparing MN soma area with Cav-1 expression.

We next assayed for lower motor neuron (MN) cell counts and morphology in the lumbar ventral horn region (L3-L5) after subpial *SynCav1* delivery (Figure 5A). As shown in Figure 5B-5E, the total number and cell body (i.e., soma) area of ChAT+ MNs were significantly decreased in both 19 wk male and female hSOD1^G93A^ naïve mice compared to WT mice, consistent with previous work that showed ALS pathology preferentially affects larger alpha motor neurons innervating faster muscle fibers 36. In contrast, *SynCav1* maintained MN numbers and normal morphology and prevented loss of spinal lumbar ventral horn MNs compared to control hSOD1^G93A^ mice. Further analysis confirmed that the decreased Cav-1 in the hSOD1^G93A^ naïve spinal cord is mostly due to the downregulated Cav-1 in the ChAT+ MNs, while* SynCav1* preserved Cav-1 expression of the ChAT+ MNs compared to hSOD1^G93A^ + control mice (Figure 5F-5G, ****p < 0.0001, Student's t-test, n = 3-8 mice/group). Noticeably, simple linear regression correlation analysis revealed a significant positive correlation between Cav-1 expression and ChAT positive MN area (Figure 5H-5I, p < 0.0001, R^2^ = 0.68). Together, these results indicate that *SynCav1* gene delivery affords significant neuroprotection in hSOD1^G93A^ spinal cord MNs in both Sexes.

**Figure 6:**
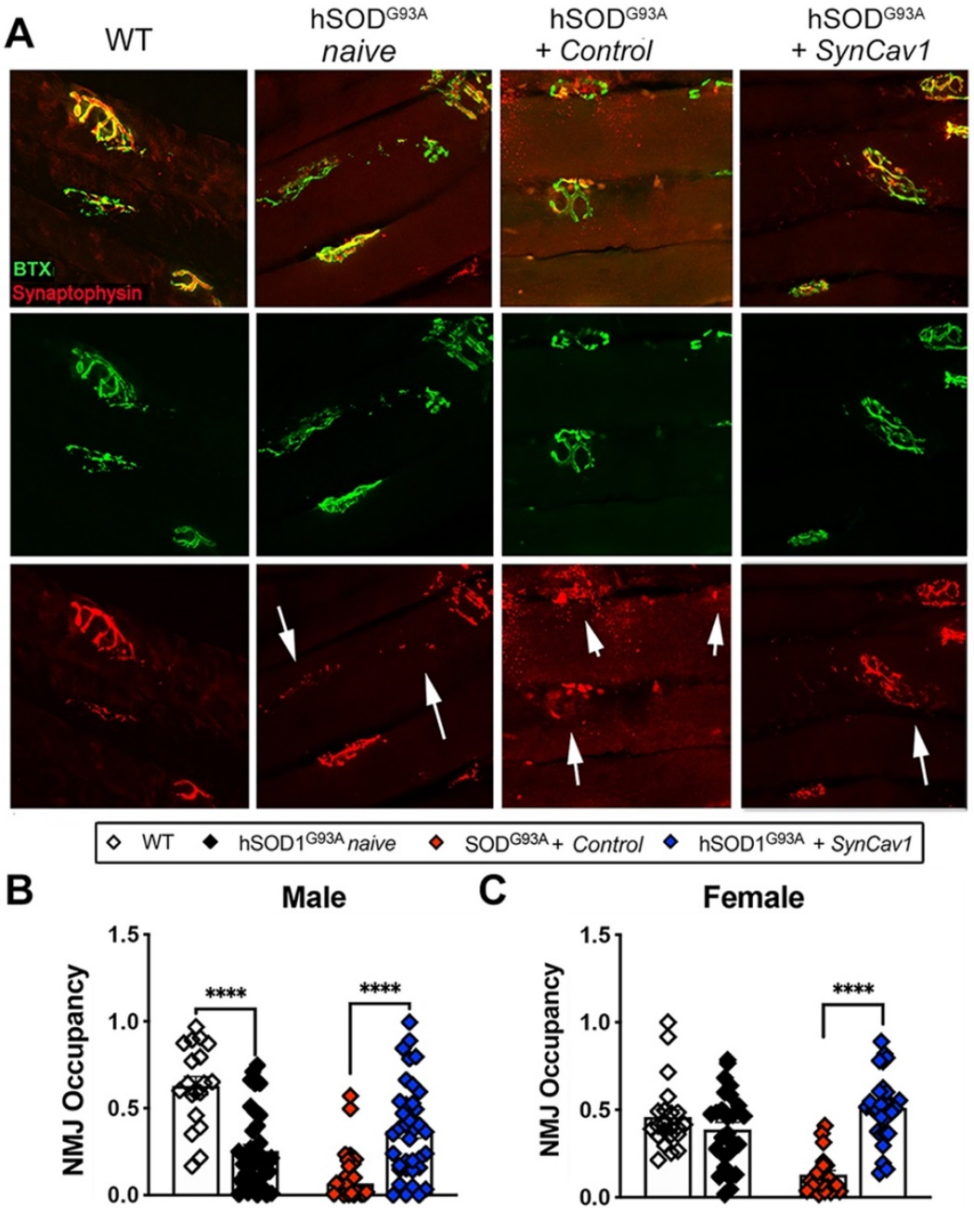
***SynCav1* delivery preserved NMJ morphology in the gastrocnemius muscle.** (A) Representative images of neuromuscular junction (NMJ) identified by bungarotoxin-alpha (Btx-α, post-synaptic) and synaptophysin (pre-synaptic vesicles). (B, C) NMJ occupancy is defined by pre-synaptic area overlaid with post-synaptic area (%). Student's t-test was performed for B and C (n = 3-5 mice/group; *p < 0.05, ****p < 0.0001).

Finally, because we observed better motor function and preservation of MNs at lower lumbar level with *SynCav1* delivery, we analyzed NMJ morphology in the gastrocnemius muscle, one of the larger lower limb muscles receiving synaptic innervation from L5-S1 spinal cord MN axons (Figure 6A). At 19 wk, mice from both hSOD1^G93A^ naïve and hSOD1^G93A^ + control exhibited a significant decrease in NMJ occupancy (Figure 6B-6C, male, ****p < 0.0001, n = 3-5 mice/group; female, ****p < 0.0001, n = 3-5 mice/group; Student's t-test), with only 30% and 20% of the NMJs occupancy measured in naïve and control hSOD1^G93A^ mice respectively, indicating severe denervation of the GA muscle 37. Noticeably, NMJ occupancy in hSOD1^G93A^ + control mice were significantly lower compared to naïve hSOD1^G93A^ mice, suggesting an effect from subpial surgery alone on NMJ integrity. In contrast, the NMJ occupancy of synaptophysin in the hSOD1^G93A^ +* SynCav1* mice was significantly greater in both sexes (****p < 0.0001, n = 3-5 mice/group; Student's t-test) compared to hSOD1^G93A^ + controls, results that in line with the greater RW performance observed in the hSOD1^G93A^ *+ SynCav1* group.

### *AAV9-SynCav1* treated hSOD1^G93A^ rats exhibit better grip strength and MEPs (larger amplitude and lower latency)

We next tested whether subpial *AAV9-SynCav1* delivery in a larger animal model (i.e., hSOD1^G93A^ rat model) could mitigate ALS-like disease onset. Transgenic negative or positive rats (Figure S2A) received control vector (WT + control or hSOD1^G93A^ + control) or *AAV9-SynCav1* hSOD1^G93A^ + *SynCav1*) by cervical (C4/C5) subpial delivery at 2 m. At 4 m (2 m post vector delivery), IB and IF of spinal cord tissue confirmed strong Cav-1 expression in MN from *SynCav1* administered rats (Figure S2B-S2C). We next measured neuromuscular function by using forelimb grip strength (GS) and MEPs in the brachial (upper forelimb) and gastrocnemius muscles (lower calf). GS and MEPs were initially measured at 8 wk (prior to subpial AAV delivery) and then measured weekly from 10-20 wk. hSOD1^G93A^ *+ SynCav1* rats exhibited significantly greater peak force (g) grip strength at 13, 14, and 15 wk compared to hSOD1^G93A^ **+** control rats (Figure S3A; n = 17-18 rats/group; **p < 0.01, ***p < 0.001, 2-way ANOVA, Bonferroni's multiple comparisons test). MEP recordings revealed significant greater amplitude (mV) at 18 wk (Figure S3B) and shorter latency (millisecond, ms) at 15 wk (Figure S3C) in the upper brachial forelimb muscle; lower limb MEP recordings revealed significant greater amplitude (mV) at 13, 14, and 15 wk (Figure S3D) and lower latency (ms) at 20 wk (Figure S3E) in the gastrocnemius muscle of hSOD1^G93A^ + *SynCav1* rats compared to hSOD1^G93A^ + control (n = 8 rats/group; *p < 0.05, **p < 0.01, ***p < 0.001, ****p < 0.0001, 2-way ANOVA, Bonferroni's multiple comparisons test). These data from hSOD1^G93A^ rats treated with *SynCav1* builds upon the findings already demonstrated in hSOD1^G93A^ mice and provides further direct evidence of the therapeutic potential properties afforded by *SynCav1* in a larger ALS animal model.

## Discussion

The present study tested a neuroprotective, non-monogenic targeting gene delivery in the hSOD1^G93A^ mouse model of ALS. These results show that subpial delivery of AAV9-*SynCav1* delays disease onset and early disease progression, preserves ventral horn spinal motor neurons in a familial mouse model of ALS, independent of reducing the toxic monogenic protein engineered to cause the ALS-like neuropathology. Delivering genes that augment neuroprotective proteins may mitigate spinal motor neurodegeneration, prolong survival in individuals afflicted with ALS. The present study showed *AAV9-SynCav1* subpial delivery significantly delays disease onset and extends survival in both male and female hSOD1^G93A^ mice, results that are comparable to previous studies that used ASOs to target mutant monogenic hSOD1 in hSOD1^G93A^ rodents 26. Importantly, *SynCav1* gene therapy proved efficacious in both sexes, independent of affecting expression of mutant hSOD1 protein. This latter finding suggests that *SynCav1* possesses the therapeutic potential to combat other forms of FALS (e.g., *SOD*, *FUS*, *C9orf72*, *TDP-43*) 6-8, 38, 39 as well as SALS attributed to unknown etiology. Additional work is needed to test whether *SynCav1* can work synergistically with other biologics or small molecules designed to knockdown known monogenic links to FALS.

A limitation of the current study is that the extended survival achieved by exogenous vector delivery is slightly lower (10% extended survival) than that observed when crossing SynCav1 transgenic mice with hSOD1^G93A^ mice (19% extended survival) 25. This discrepancy between the two studies may be attributed to the following reason: while SynCav1 TG mice overexpress neuronal Cav-1 throughout the whole CNS, the physical and anatomical restrictions of lumbar subpial *AAV9-SynCav1* delivery in the small rodent limits broad CNS tissue AAV9 distribution and transduction and subsequent Cav-1 transgene expression. As shown in Figure 1 and Figure S1, Cav-1 transgene expression with *SynCav1* predominantly distributed in the lumbar cord (~ 4-fold) and to a less degree in the thoracic cord (~ 2-fold), with no over-expression in the cervical cord after subpial delivery. Failure to target the cervical spinal cord 14, specifically the cervical plexus (C3-C5) which projects motor innervation necessary to regulate diaphragm muscle function, most likely restricted our ability to mitigate respiratory failure in these hSOD1^G93A^ mice, the most common cause of death from ALS. However, even with this limitation of achieving cervical AAV9 spinal cord distribution/transduction and subsequent Cav-1 transgene expression, the therapeutic efficacy afforded by subpial *SynCav1* gene delivery is comparable to approaches targeting the known monogenic cause of the mutant SOD1 neuropathology 40. For example, a recent study by Powell et al. 2022 18 delivered their AAV at wk 8 (similar time point in the current study) using CRISPR technology (AAV-RfxCas13d) to reduce mutant hSOD1 by 65% throughout the entire spinal cord (cervical to lumbar) of hSOD1^G93A^ mice, which demonstrated a similar extension of survival. A combined therapy consisting of *SynCav1* with approaches targeting knockdown of monogenic causes of ALS may work in concert to treat ALS more effectively.

On a cellular level, Cav-1 regulates cellular endocytosis and vesicular trafficking 41-43, subcellular localization of NTRs and other synaptic signaling components 11, 12, 29, preserves mitochondrial function in the brain of AD (PSAPP) mice 44 and mitochondrial morphology in the spinal cord of ALS (hSOD1^G93A^) mice 25. It is well known that mutations in several genes linked to ALS (e.g., Tar DNA binding protein gene (TARDBP) which encodes for TDP-43 6, fused in sarcoma (*FUS*) 39, and the C9 open-reading frame 72 genes (*C9orf72*) 7, 8) impair axonal transport and alter mitochondrial function and dynamics (fission/fusion and trafficking) 9, 10, 45, 46. Although not investigated in the present study, the neuronal protection and preservation afforded by *SynCav1* may in part be through the maintenance/restoration of functional axonal transport of cellular cargoes such as NTR signaling endosomes 47, 48 and mitochondria 49. Using novel *in vitro* model systems consisting of human motor neurons co-cultured with human myotubes in specialized microfluidic chambers 50-53 exposed to neurotoxic dipeptide repeats (DPRs) commonly found in FALS 8, will allow the ability to measure axonal transport of organelles and key cellular cargoes 9, 49, 54, 55 to better elucidate the molecular mechanisms underlying the neuroprotective properties of *SynCav1*.

Preservation of NMJ morphology is essential for preserving motor function and life quality. NMJs are unique synapses that facilitate communication between MNs and the effector muscles. Due to their long axons, MNs are extremely susceptible to retraction during pathological conditions such as ALS, thus resulting in disrupted communication between the MN and effector muscles. Initially, the MNs try to compensate for the retraction by inducing axonal sprouting and collateral innervation. However, with disease progression, this compensation fails and results in final MN death, a neuropathological event known as the “dying back” mechanism 56. The subsequent NMJ disruption causes significant skeleton muscle atrophy, respiratory insufficiency, and death 57, 58. The present study revealed that *AAV9-SynCav1* preserved NMJ integrity and improved running wheel performance in both* hSOD1^G93A^* male and female mice. Could *SynCav1* be applied at this MN 'compensatory stage' to prevent or overcome the “dying back” mechanism and maintain functional NMJ synapses? As noted above, the use of specialized *in vitro* models consisting of human motor neurons forming functional NMJs with human myotubes could optimize the temporal window at which to deliver *SynCav1* in the presence of otherwise toxic dipeptide species to overcome this “dying back” cellular event. Another limitation to the current study is the lack of more sensitive neuromuscular measurements of functional NMJ physiology in the upper or lower limbs (e.g., MEPs, grip strength) performed specifically in the hSOD1^G93A^ mouse ALS model. However, using the larger hSOD1^G93A^ rat ALS model, subpial cervical spinal cord *SynCav1* delivery resulted in significantly better forelimb grip strength and MEPs (greater amplitude and decreased latency) in both upper limb brachial and lower limb gastrocnemius muscles, further evidence of *SynCav1's* efficacy using two different ALS model species.

The current study delivered *SynCav1* at week 8 (pre-symptomatic stage) to the lumbar cord which exhibited similar folds increase as observed in the healthy wild type mice. However, vector dosing needed to achieve efficacious transgene expression levels may depend upon disease stages 59. If vector is delivered at a later stage, Cav-1 expression in the lumbar cord neuron may fail to achieve the same level of fold increase since the neurodegenerative spinal cord may not hold the capacity to express an exogenous transgene to the same levels as healthy spinal cord, an important pre-clinical finding to consider when formulating human equivalent dosing to achieve clinical efficacy 60-63. Interestingly, we measured adverse effects from the subpial surgery on BW at the late stage of disease compared to the naïve hSOD^G93A^ mice, with no effect on disease onset or survival. A possible explanation is that the surgery may have exacerbated back muscle weakness, which in turn could have affected the ability to retrieve food located on top of the cage for late stage hSOD1^G93A^ mice. This inability to retrieve food would result in a more rapid BW decline compared to naïve hSOD1^G93A^ mice. The fact that we did not measure any difference in survival, MN morphology or MN count between hSOD1^G93^ control and hSOD1^G93^ naïve mice, indicate that the adverse effect from subpial surgery on BW is not through affecting MN survival directly. Nevertheless, since the lumbar subpial surgery involves an invasive laminectomy, which is difficult to perform successfully on older, severely symptomatic hSOD1^G93A^ mice. Exploration of alternative routes of *SynCav1* administration (i.e., intrathecal and intracerebroventricular) are worthy of investigation.

In summary, the present study demonstrates that subpial *SynCav1* gene delivery to the lumbar spinal cord of hSOD^1G93A^ mice prolongs survival and maintains body weight in both males and females, preserves alpha motor neurons in the ventral horn with a direct correlation to soma size and Cav-1 expression, and preserves NMJs in the lower limbs, all of which occurred independent of affecting expression of mutant hSOD1. These data suggest that *SynCav1* might serve as a novel gene therapy for neurodegenerative conditions in ALS and other forms of CNS disease of unknown etiology. Further studies are needed to determine the effect of *SynCav1* on disease progression when delivered at later stages of the disease.

## Supplementary Material

Supplementary figures.

## Author contributions

S.W. guided the overall study design, writing the manuscript, IB and IF microscopy; T.I. performed subpial gene delivery, behavioral tests and IF microscopy; P.S. and D.W. assisted with animal breeding and behavior tests; A.S. performed all rat subpial vector delivery and motor tests; X.L, T.D., and W.L assisted with data analysis; J.A.B. and E.K. assisted with editing of the manuscript; A.M. generated AAV vectors (UCSD Viral Vector Core); D.M.R., H.H.P. and P.M.P. assisted with manuscript editing; T.T and M.M. provided subpial surgical guidance; B.P.H. guided the overall study design, scientific concept, edited the manuscript, and funded the project (VA SPiRE award 5I21RX003324).

## Ethics approval

All animal protocols were approved by the Veterans Administration San Diego Healthcare System Institutional Animal Care and Use Committee (#A20-030).

## Abbreviations

ALS: Amyotrophic lateral sclerosis
AAV9: adeno-associated virus serotype 9
Btx-α: bungarotoxin-α
ChAT: choline acetyltransferase
FALS: familial ALS
GS: grip strength
SALS: sporadic ALS
SOD: super oxide dismutase
SynCav1: synapsin-promoted caveolin-1 genetic construct
MEPs: motor evoked potentials
MLRs: membrane/lipid rafts
MN: motor neuron
NMJ: neuromuscular junction
