# Supplementary material for: Subpial delivery of adeno-associated virus 9-synapsin-caveolin-1 (AAV9-SynCav1) preserves motor neuron and neuromuscular junction morphology, motor function, delays disease onset, and extends survival in hSOD1G93A mice
Source: Theranostics. 2022 Jul 11;12(12):5389–403. doi: 10.7150/thno.72614 (PMC9330519; doi:10.7150/thno.72614)
Supplement: Supplementary file 1 — Supplementary figures. [file thnov12p5389s1.pdf]

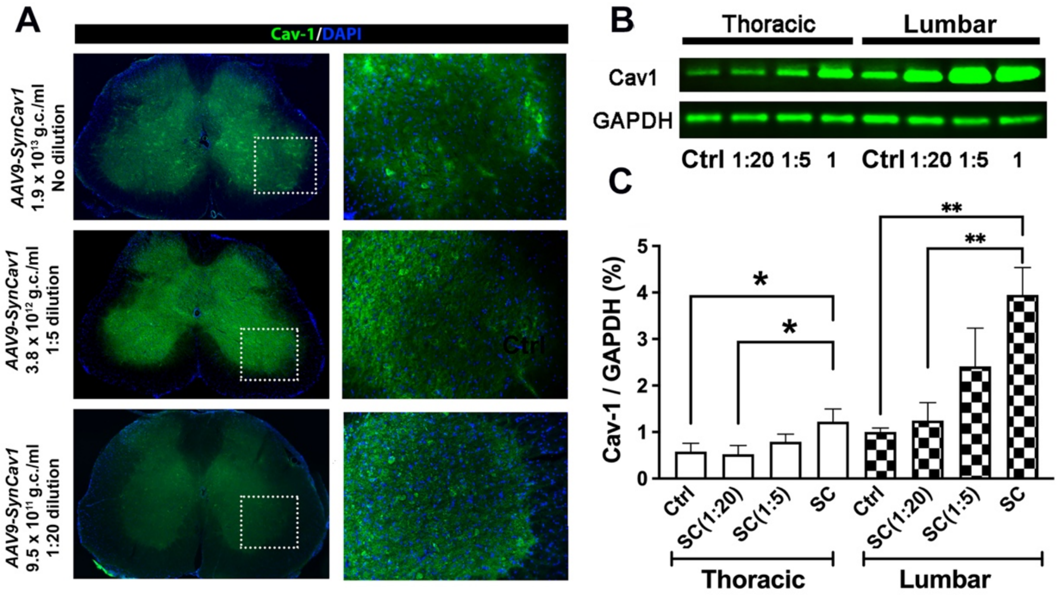

Figure S1. Dose effect of subpial AAV9-*SynCav1* delivery at L1 spinal cord in WT mice. Three ascending doses (10 μl of 9.5×10^{11}, 3.8×10^{12}, 1.9×10^{13} genome copies (GC)/ml) were delivered by subpial administration. (A) Cav-1 immunofluorescence within L3-L4 spinal cord regions 1 month-post subpial AAV9-*SynCav1* (SC) delivery. (B) Immunoblot of Cav-1 at lumber and thoracic segments are shown. (C) Densitometry analysis was expressed as mean ± S.E.M. Unpaired Student's t-test (n = 3 mice/group; $*p < 0.05$, $**p < 0.01$).

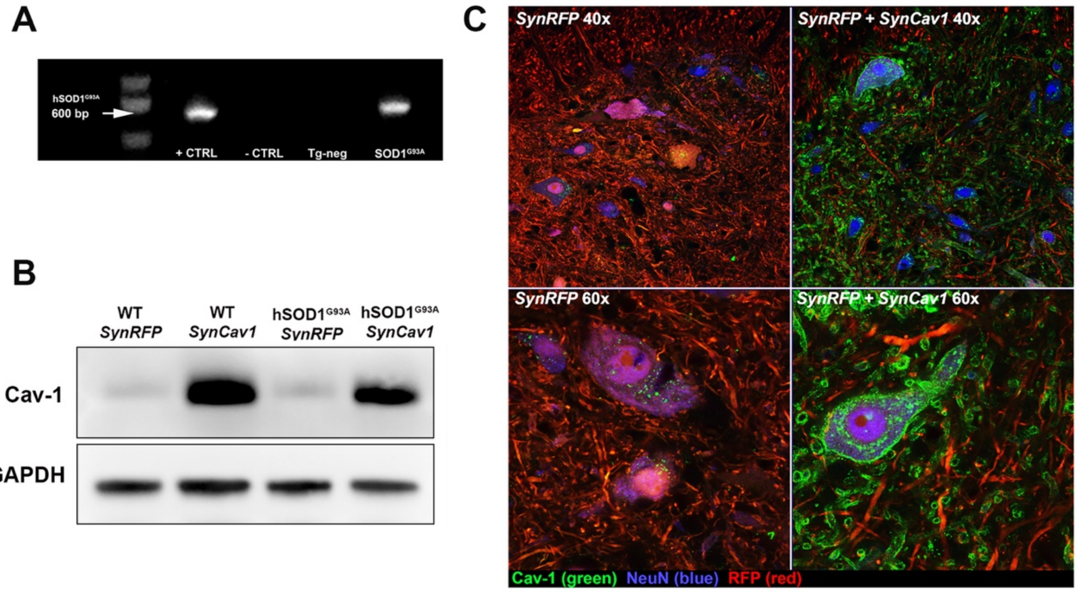

Figure S2. Cervical subpial *AA9-SynCav1* delivery increases Cav-1 protein expression in the spinal cord of hSOD1^{G93A} rats. (A) Genotyping of rat (28 d) containing hSOD1^{G93A}. Gel electrophoresis of PCR products for human SOD1 (h, 600 bp, +CTRL), -CTRL, transgene negative rat tissue (Tg-neg), and rat hSOD1^{G93A} transgenic (600 bp). (B) Representative Cav-1 and GAPDH immunoblots of spinal cord tissue homogenates from wild type (WT) or hSOD1^{G93A} rats that received subpial *AAV9-SynRFP* or *AAV9-SynCav1* at 60 d. Tissue was harvested 2 m post vector delivery (120 d old rats). (C) Representative images of cervical sections from animals that received cervical subpial delivery of *AAV9-SynRFP* only or *AAV9-SynRFP* with *AAV9-SynCav1*.

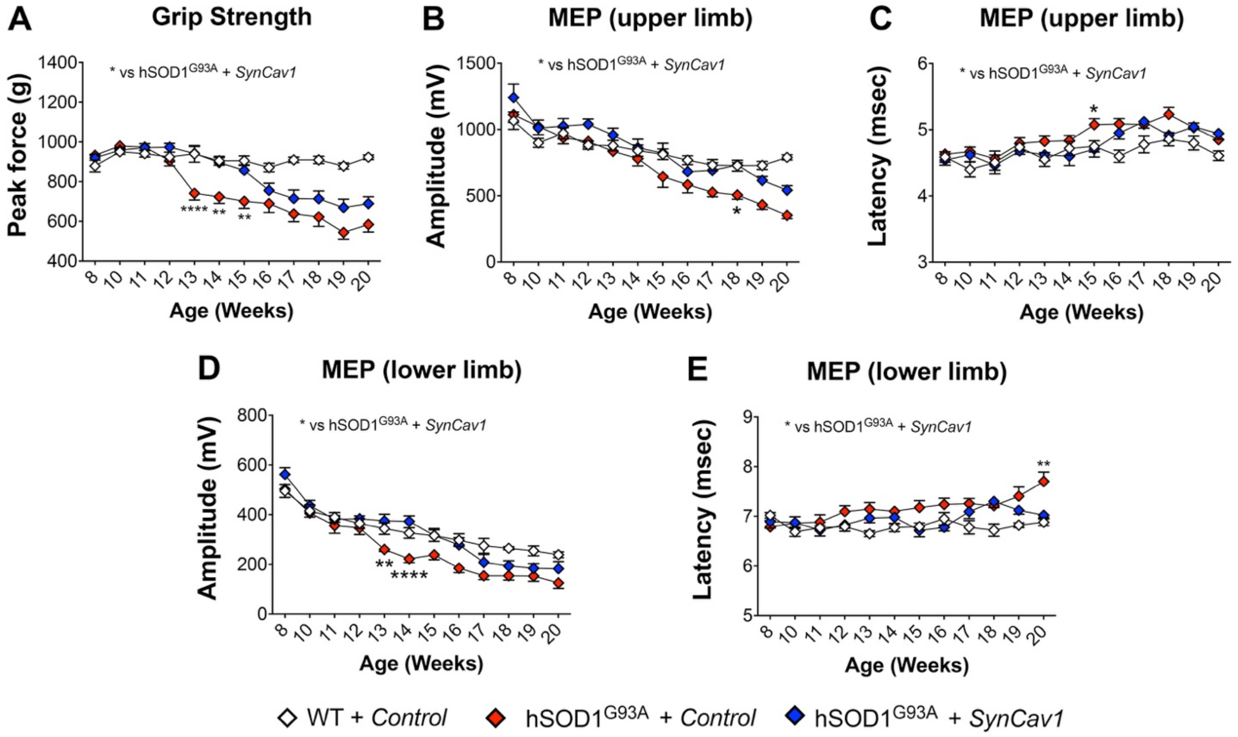

Figure S3. Cervical subpial *AA9-SynCav1* to hSOD1^{G93A} rats preserves grip strength and MEPs in upper (brachial) and lower (gastrocnemius) muscles. WT (transgene negative) or hSOD1^{G93A} rats received control vector or *AAV9-SynCav1* by subpial cervical delivery at 60 d. (A) Forelimb grip strength, (B) upper limb (brachial) amplitudes (mV), (C) brachial limb latency (msec) recordings, (D) lower limb (gastrocnemius) amplitude (mV), and (E) lower limb (gastrocnemius) latency (msec) were measured at 8 wk (prior to AAV delivery) and weekly from 10 wk (post AAV delivery) to 20 wk. Data are expressed as mean ± SEM. Two-way ANOVA with Bonferroni's multiple comparisons post hoc test (n = 17-18 rats/group for grip strength; n = 8 rats/group for MEPs; *$p < 0.05$, **$p < 0.01$, ***$p < 0.005$, ****$p < 0.0001$
